# Clinical and pathological features of anti-glomerular basement membrane disease associated with membranous nephropathy: an observational study

**DOI:** 10.1080/0886022X.2022.2141645

**Published:** 2022-11-09

**Authors:** Shasha Zhang, Chaofan Li, Jing Huang, Yan Zhou, Caifeng Gao, Mengyao Sun, Rong Wang, Bing Chen

**Affiliations:** a Department of Nephrology, Shandong Provincial Hospital Affiliated to Shandong First Medical University, Jinan, China; b Department of Nephrology, Shandong Provincial Hospital, Cheeloo College of Medicine, Shandong University, Jinan, China; c Department of Nephrology, Jinan Shizhong People’s Hospital, Jinan, China

**Keywords:** Anti-glomerular basement membrane disease, membranous nephropathy, crescents, prognosis

## Abstract

To investigate the clinical manifestations, pathological features, pathogenesis, treatment, and prognosis of anti-glomerular basement membrane (anti-GBM) disease with membranous nephropathy (MN). Seven patients with anti-GBM disease and concurrent MN were enrolled in this study. Control subjects included 13 patients with anti-GBM glomerulonephritis (GN) and 6 with anti-GBM disease and concurrent anti-neutrophil cytoplasmic antibodies-associated disease (anti-GBM + ANCA). Laboratory tests and pathological information were analyzed before immunosuppressive therapy or plasmapheresis administration. Prognosis was assessed in continuous follow-up. In the anti-GBM + MN group, 28.57% of patients exhibited acute kidney disease, lower than that in the anti-GBM GN group (84.62%, *p* = .022). None of the anti-GBM + MN or + ANCA patients exhibited hemoptysis, but 15.4% of anti-GBM GN patients did, with no significant difference (*p* = .720). Only 14.3% of anti-GBM + MN patients had crescentic GN. The proportion of necrosis averaged 29.0% in the anti-GBM + MN group. Survival curve analysis revealed that renal outcomes in the anti-GBM + MN group were better than those in the anti-GBM GN group (*p* = .019). Patients with both anti-GBM disease and MN showed atypical anti-GBM GN. They had a lower proportion of glomerular crescents and a better renal function prognosis than patients with classical anti-GBM GN. To improve renal recovery, early identification and treatment of anti-GBM disease associated with MN is needed.

## Introduction

Anti-glomerular basement membrane (anti-GBM) disease is an autoimmune disease associated with anti-GBM autoantibodies, characterized by rapidly progressive glomerulonephritis (GN) or alveolar hemorrhage. The noncollagenous domain of the α3 chain of type IV collagen (α3(IV)NC1) on the GBM is the main antigen [1]. Although the initiating mechanism is unclear, many factors, such as exposure to chemical solvents, smoking, urinary tract infection, and renal trauma, are related to its pathogenesis [2–4]. Anti-GBM disease presents as a monophasic course with a low recurrence rate [5,6]. However, rapid decline in renal function and severe pulmonary hemorrhage can substantially increase mortality. Therefore, early identification and treatments are recommended to partially reverse renal damage and avoid lifelong dependence on dialysis.

Several cases of anti-GBM disease associated with other nephropathies have been reported. For example, 21%–47% of patients with anti-GBM disease are anti-neutrophil cytoplasmic antibody (ANCA) positive, with anti-myeloperoxidase (MPO) antibodies being more frequent than anti-proteinase 3 antibodies [7–10]. Since the initial report in 1974, only a few case series of anti-GBM disease associated with membranous nephropathy (anti-GBM + MN) have been described [11–13]. This study focused on the clinical manifestations, pathological features, treatment, and prognosis of anti-GBM + MN to offer insight into its pathogenesis and early diagnosis as well as therapeutic schemes.

## Materials and methods

We analyzed 7 patients with anti-GBM + MN from January 2010 to January 2021 in Shandong Provincial Hospital. Demographic, clinical, laboratory, and pathological information for 13 patients with anti-GBM disease and 6 patients with ANCA-positive anti-GBM disease (anti-GBM + ANCA) were also collected before immunosuppressive therapy or plasmapheresis (PP) was initiated. Follow-up information was obtained from medical record reviews and telephone surveys.

Anti-GBM disease was diagnosed by circulating or renal anti-GBM antibody positivity. Furthermore, the key diagnostic criteria of anti-GBM GN was a large proportion of crescents at similar stages, with linear staining of IgG along the GBM [14]. Patients who tested positive for ANCAs were enrolled in the anti-GBM + ANCA group. All the ANCA-positive patients in our study were anti-MPO positive. Renal samples with anti-GBM disease were involved in the anti-GBM + MN group if they also displayed glomerular capillary wall thickening, granular deposition of IgG and C3 along the capillary wall, and glomerular subepithelial electron-dense deposition. The causes which could be associated with secondary MN, such as hepatitis B/C virus infection, lupus, malignancy, drugs, and heavy metal poisoning, were not found in our anti-GBM + MN cases. Considering extracorporeal shock wave lithotripsy (ESWL) may be associated with anti-GBM pathogenesis, we focused on the history of renal stones and ESWL in the medical investigation [15]. No subjects had renal calculi or accepted ESWL prior to the diagnosis. In addition, no stones were found by renal ultrasound at the time of diagnosis.

Intensity of immunofluorescence staining of immunoglobulin, complement and target antigen was semiquantitatively scored as five grades (−, ±, +, ++, and +++). The crescents involved at least 10% of the circumference of the Bowman and were constituted by at least two layers of cells. The segmental forms (small crescents) involved less than 50% of the circumference and the global forms (large crescents) involved more than 50% of the circumference. The crescentic GN was defined as large crescents formation in more than 50% of glomeruli [16].

Circulating anti-PLA2R antibodies were detected by enzyme-linked immunosorbent assay using anti-PLA2R IgG kit (EA1254-9601G, EUROIMMUNE, Germany). The operation procedure was in strict accordance with the standard process of the manual and described previously [17]. The positive cutoff value was ≥20 RU/mL. Immunohistochemical staining was used to detect the PLA2R antigen in paraffin sections of renal tissues. The specific primary antibody, anti-PLA2R antibody, was purchased from Abcam at a dilution ratio of 1:100. Positive glomerular PLA2R expression was defined as coarse granular staining with different intensities along the capillary ring as described previously [18].

The estimated glomerular filtration rate (eGFR) was calculated based on serum creatinine (SCr) levels using the Chronic Kidney Disease Epidemiology Collaboration equation [19]. Acute kidney injury (AKI) was defined as SCr rising by over 50% from baseline within 7 days or more than 26.5 μmol/L within 2 days or urine volume (UV) less than 0.5 mL/kg/h for 6 h. Acute kidney diseases and disorders (AKD) included AKI and conditions of renal impairment (eGFR < 60 mL/min/1.73 m^2^ or decreasing by ≥ 35% or SCr increasing by > 50%) or structural damage indicators lasting for less than 3 months. End-stage renal disease (ESRD) referred to a status involving dependency on dialysis for more than 3 months during follow-up or before death. Renal survival period ended when patients entered ESRD stage.

This retrospective study conformed to the Declaration of Helsinki. The Ethics Committee of Shandong Provincial Hospital (LCYJ: No. 2019-105) approved the research and waived informed consent.

SPSS software, version 26.0 (IBM, Inc., USA) was used for statistical analysis. Normal data were presented as the mean ± standard deviation; nonnormal data were presented as the median and quartile. A *t* test was applied to assess differences in normally distributed data. Differences in nonnormal data were verified by the Kruskal–Wallis test or Mann–Whitney U test. Qualitative data were compared by using Fisher’s exact test. Renal function outcomes were analyzed by Kaplan–Meier survival curves and log-rank tests. The significance level (*p* value) was set at < .05 (two-tailed).

## Results

### Comparison of clinical and laboratory features

As shown in Table 1, patients in the three groups were mainly older than 45 years old (anti-GBM + MN 85.7%, anti-GBM + ANCA 83.3%, and anti-GBM GN 69.2%), with no significant difference in age (*p* = .764). All anti-GBM + ANCA patients were female. No significant sex difference existed between the other two groups (*p* = .642).

**Table 1.** Comparisons of clinical and laboratory features of anti-GBM disease patients with and without MN or ANCA.

	Anti-GBM + MN (*n* = 7)	Anti-GBM + ANCA (*n* = 6)	Anti-GBM GN (*n* = 13)	*p* value
Age (years)	54.0 (52.0, 65.0)	61.5 (37.3, 73.0)	60.0 (32.5, 65.0)	.764
Sex, % (male)	42.9 (3/7)	0.0 (0/6)	61.5 (8/13)	.045^a^
Onset to diagnosis, months	12.0 (0.5, 24.0)	0.5 (0.2, 4.0)	1.0 (0.7, 2.0)	.102
AKD, % (*n*)	28.57 (2/7)	83.33 (5/6)	84.62 (11/13)	.030^b^
Smoking, % (*n*)	0.0 (0/7)	16.7 (1/6)	23.1 (3/13)	.647
Hemoptysis, % (*n*)	0.0 (0/7)	0.0 (0/6)	15.4 (2/13)	.720
Pulmonary infection, % (*n*)	0.0 (0/7)	50.0 (3/6)	30.8 (4/13)	.132
Hemoglobin (g/L)	104.14 ± 23.30	74.17 ± 21.56	86.38 ± 14.88	.028^c^
Serum creatinine (μmol/L)	183.00 (92.40, 763.00)	699.30 (269.75, 1124.20)	357.40 (150.30, 513.15)	.363
eGFR (mL/min/1.73m^2^)	33.00 (6.00, 66.00)	6.00 (3.00, 16.25)	18.00 (8.50, 42.50)	.133
Serum albumin (g/L)	26.5 (21.9, 28.4)	32.95 (30.43, 34.38)	28.50 (23.55, 30.85)	.046
Serum C3 (g/L)	1.52 ± 0.24	1.01 ± 0.31	1.16 ± 0.19	.004^d^
Serum C4 (g/L)	0.37 ± 0.09	0.29 ± 0.05	0.27 ± 0.05	.099
Anti-GBM antibodies (RU/mL)	156.70 (114.30, 1437.80)	295.03 (91.01, 582.78)	158.90 (112.06, 237.00)	.843
Positive rate of anti-PLA2R^e^, %	66.7 (2/3)	0.0 (0/3)	0.0 (0/2)	–
Anti-MPO antibodies (RU/mL)	All negative (<20)	112.82 ± 63.83	All negative (<20)	–
Oliguria/anuria, % (*n*)	0.0 (0/7)	50.0 (3/6)	38.5 (5/13)	.115
Gross hematuria, % (*n*)	28.6 (2/7)	16.7 (1/6)	15.4 (2/13)	.817
Urinary erythrocyte (HPF)	22.30 (7.83, 406.36)	116.80 (53.88, 333.75)	133.20 (24.85, 332.05)	.477
Urinary protein (g/24 h)	1.08 (0.40, 2.64)	1.52 (1.29, 1.82)	1.44 (0.55, 3.50)	.843

GBM: glomerular basement membrane; GN: glomerulonephritis; MN: membranous nephropathy; ANCA: antineutrophil cytoplasmic antibodies; AKD: acute kidney diseases and disorders; eGFR: estimated glomerular filtration rate; PLA2R: phospholipase A2 receptor; MPO: myeloperoxidase; HPF: high-power field.

^a^Anti-GBM + ANCA versus anti-GBM GN, *p* = .018.

^b^Anti-GBM + MN versus anti-GBM GN, *p* = .022.

^c^Anti-GBM + MN versus anti-GBM + ANCA, *p* = .027.

^d^Anti-GBM + MN versus anti-GBM + ANCA, *p* = .004. anti-GBM + MN versus anti-GBM GN, *p* = .023.

^e^The cutoff value of anti-PLA2R was 20 RU/mL; the positive rate was calculated among patients with data available.

The proportion of AKD in the anti-GBM + MN group reached 28.57% (2/7), which was lower than that in the anti-GBM disease group (84.62%, 11/13, *p* = .022). Patients in the three groups had a lower proportion of smoking history, with 0.0% (0/7) in the associated MN group, 16.7% (1/6) in the + ANCA group, and 23.1% (3/13) in the anti-GBM GN group, though the difference was not statistically significant (*p* = .647). None of the patients in the anti-GBM + MN or + ANCA groups exhibited hemoptysis, but 15.4% (2/13) of anti-GBM GN patients did, also with no significant difference (*p* = .720).

Baseline levels of eGFR decreased in all three groups, 33.00 (6.00, 66.00) mL/min/1.73 m^2^ in the group with MN, 6.00 (3.00, 16.25) mL/min/1.73 m^2^ in the group with ANCA, and 18.00 (8.50, 42.50) mL/min/1.73 m^2^ in the anti-GBM GN group, with no significant difference (*p* = .133). There was also no significant difference in SCr among the three groups (*p* = .363), with median levels of 183.00 μmol/L in the anti-GBM + MN group, 699.30 μmol/L in the anti-GBM + ANCA group, and 357.40 μmol/L in the anti-GBM GN group. Mean levels of baseline serum complement were within the normal range in all the groups. However, the C3 level was higher in the anti-GBM + MN group (1.52 ± 0.24 g/L) than in the anti-GBM + ANCA group (1.01 ± 0.31 g/L, *p* = .004) and the anti-GBM disease group (1.16 ± 0.19 g/L, *p* = .023).

Levels of anti-GBM antibodies were higher than the normal range in the three groups, but there was no significant difference among them (*p* = .843). Limited by the technology available at onset, only some patients were tested for anti-phospholipase A2 receptor (PLA2R) antibodies; positive rates were 66.7% (2/3) in the associated MN group, 0.0% (0/3) in the + ANCA group, and 0.0% (0/2) in the anti-GBM GN group.

The median level of urinary erythrocytes in the anti-GBM + MN group was lower (22.30/HPF) than that in the other two groups (116.80/HPF in the anti-GBM + ANCA and 133.20/HPF in the anti-GBM GN groups), but the difference was not significant (*p* = .477). The three groups of patients showed varying degrees of proteinuria, with a median level of 1.08 g/24 h in the associated MN group, 1.52 g/24 h in the + ANCA group, and 1.44 g/24 h in the anti-GBM GN group. There was no significant difference in urinary protein among them (*p* = .843).

### Pathological characteristics of anti-GBM + MN

Seven patients with anti-GBM + MN were included in this study. Their renal biopsy pathological information is shown in Table 2. Under light microscopy, 71.4% (5/7) and 28.6% (2/7) of anti-GBM + MN pathological samples were in stage I and stage II MN, respectively. A total of 28.6% (2/7) also displayed acute tubular necrosis. On average, 27 glomeruli were observed per sample, and 43.4% of glomeruli had crescents. A total of 14.3% (1/7) of the patients had crescentic GN. The proportion of global sclerosis averaged 9.7%; the proportion of necrosis averaged 29.0%.

**Table 2.** The pathological features of patients with anti-GBM + MN.

Case	1	2	3	4	5	6	7
Light microscopy pattern	MN + crescents	MN + crescents + ATN	MN + crescents	MN + crescents	MN + crescents	MN + crescents + ATN	MN + crescents
Routine staining	IgG 3+, IgA–, IgM–, C3 2+, C1q 1+	IgG 3+, IgA 1+, IgM 2+, C3 2+, C1q 2+	IgG 3+, IgA–, IgM 1+, C3 3+, C1q 2+	IgG 3+, IgA–, IgM–, C3 2+, C1q–	IgG 3+, IgA 2+, IgM 1+, C3 2+, C1q 2+	IgG 3+, IgA–, IgM 1+, C3 1+, C1q 1+	IgG 3+, IgA–, IgM–, C3 1+, C1q–
IgG subtype	IgG1 1+, IgG2–, IgG3 1+, IgG4 3+	NA	IgG1–, IgG2–, IgG3–, IgG4 1+	IgG4 1+	IgG4 1+	IgG1 3+, IgG4 1+	IgG4 1+
Light chain staining	κ 3+, λ 3+	NA	κ 3+, λ 3+	κ 2+, λ 3+	κ 3+, λ 3+	κ 2+, λ 2+	κ 3+, λ 3+
Pattern of deposits	Granular capillary wall	Granular capillary wall	Granular capillary wall	Granular capillary wall	Granular capillary wall. IgG: liner 1+.	Granular and linear capillary wall	Granular capillary wall
PLA2R staining	Negative	NA	1+	1+	1+	1+	Negative
Global sclerosis	0.0% (0/37 G)	0.0% (0/10 G)	5.3% (2/38 G)	9.4% (3/32 G)	20.0% (5/25 G)	25.0% (3/12 G)	8.3% (3/36 G)
Necrosis	45.9% (17/37 G)	0.0% (0/10 G)	55.3% (21/38 G)	18.8% (6/32 G)	8.0% (2/25 G)	75.0% (9/12 G)	0.0% (0/36 G)
Glomerular crescents	32.4% (12/37 G): 3LCC and 9SCC	50.0% (5/10 G): SCC + SFCC	50.0% (19/38 G): 15 LCC + LFCC and 4 SCC	37.5% (12/32 G): LCC	28.0% (7/25 G): LCC	75% (9/12 G): 3LCC, 5LFCC, and 1LFC	30.6% (11/36 G): LCC
Mesangial proliferation	No	Yes	No	No	No	Yes	No
RBC casts	Yes	Yes	Yes	Yes	Yes	No	No
Tubular atrophy	20%	15%	20%	50%	10%	30%	10%
Interstitial edema/fibrosis	20%	15%	20%	40%	20%	20%	10%
Inflammatory cells infiltration	Lymphocytes; monocytes; plasmocytes; neutrophils; eosinophile granulocytes	Lymphocytes	Lymphocytes; monocytes; plasmocytes; neutrophils; eosinophile granulocytes	Lymphocytes; monocytes; neutrophils	Lymphocytes; monocytes	Lymphocytes; monocytes	Lymphocytes; monocytes; neutrophils
Arteriosclerosis	No	No	No	Segmental	Segmental	No	No
Stage of MN	I	I	II	I	I	I	II
Foot process effacement	Diffuse	NA	Diffuse	Diffuse	Diffuse	Diffuse	Diffuse
Electron-dense deposits	Subepithelial	NA	None	Subepithelial	Subepithelial	Subepithelial + GBM	Subepithelial

GBM: glomerular basement membrane; GN: glomerulonephritis; MN: membranous nephropathy; ATN: acute tubular necrosis; NA: not available; G: glomeruli; PLA2R: phospholipase A2 receptor; LCC: large cellular crescents; SCC: small cellular crescents; SFCC: small fibrocellular crescents; LFCC: large fibrocellular crescents; LFC: large fibrous crescents; RBC: red blood cell.

IgG staining was positive in all samples and 28.6% (2/7) were both linearly and granularly distributed along the glomerular capillary loops. IgA staining was positive in 28.6% (2/7) and IgM staining in 57.1% (4/7). C3 staining of 14.3% (1/7) samples showed a linear distribution. C1q staining varied in intensity. In six of these cases, staining for IgG subtype was performed. The positivity rate for IgG4 was 100.0% (6/6) and that for PLA2R was 66.7% (4/6). Under electron microscopy, 16.7% (1/6) had both subepithelial and intrabasal electron-dense deposits.

### Treatment and prognosis

As shown in Table 3, all seven patients with anti-GBM + MN showed various declines in eGFR at onset, and 85.7% (6/7) of them also had proteinuria. Nevertheless, higher anti-GBM autoantibody level between individuals or before and after treatment was not associated with worse eGFR. Anti-GBM + MN patients were treated with long-term therapy combining oral glucocorticoids (GCs) with intravenous cyclophosphamide (CYC). A total of 85.7% (6/7) of patients received intravenous methylprednisolone pulse therapy, and 71.4% (5/7) underwent PP. A total of 57.1% of patients received pulsed methylprednisolone and PP during their first hospitalization and were then maintained with oral prednisone and regular CYC injection. Regardless of the specific regimen, anti-GBM levels decreased in 100% (5/5) of patients, SCr in 71.4% (5/7), and urinary protein in 57.1% (4/7).

**Table 3.** Clinical characteristics of patients with anti-GBM + MN.

No.	At presentation						Treatment	At follow-up				
	Age, year	Sex	Anti-GBM, RU/mL	SCr, μmol/L	eGFR, mL/min/1.73 m^2^	Proteinuria, g/24 h		Duration, month	Anti-GBM, RU/mL	SCr, μmol/L	eGFR, mL/min/1.73 m^2^	Proteinuria, g/24 h
1	56	M	1437.80	92.00	79.00	8.38	GCs + Pulse + PP + CYC	15.1	↓ Neg.	↑ 102.40	↓ 70.00	↓ 1.62
2	52	F	118.08	219.00	28.00	2.64	GCs + Pulse + PP + CYC	9.6	↓ Neg.	↓ 90.00	↑ 63.00	↓ 1.76
3	65	M	1440.20	183.00	33.00	1.85	GCs + PP + CYC	15.7	↓ Neg.	↓ 99.10	↑ 68.00	↓ 0.25
4	52	F	85.60	87.00	66.00	0.40	GCs + Pulse + CYC	4.7	NA	↑ 156.00	↓ 32.00	↑ 0.72
5	54	F	156.70	841.00	4.00	1.08	GCs + Pulse + CYC	7.1	NA	↓ 111.00	↑ 48.00	↑ 1.30
6	67	M	215.50	763.00	6.00	0.10	GCs + Pulse + PP + CYC	2.8	↓ Pos.	↓ 653.00	↑ 7.00	↑ 0.20
7	20	F	114.30	154.00	41.00	0.69	GCs + Pulse + PP + CYC	9.6	↓ Neg.	↓ 53.00	↑ 131.00	↓ 0.58

GBM: glomerular basement membrane; GN: glomerulonephritis; MN: membranous nephropathy; M: male; F: female; SCr: serum creatinine; eGFR: estimated glomerular filtration rate; GCs: glucocorticoids; Pulse: pulsed methylprednisolone; PP: plasmapheresis; CYC: cyclophosphamide; Neg: negative; Pos: positive; NA: not available.

↓: decreased. ↑: increased.

**Figure 1.**
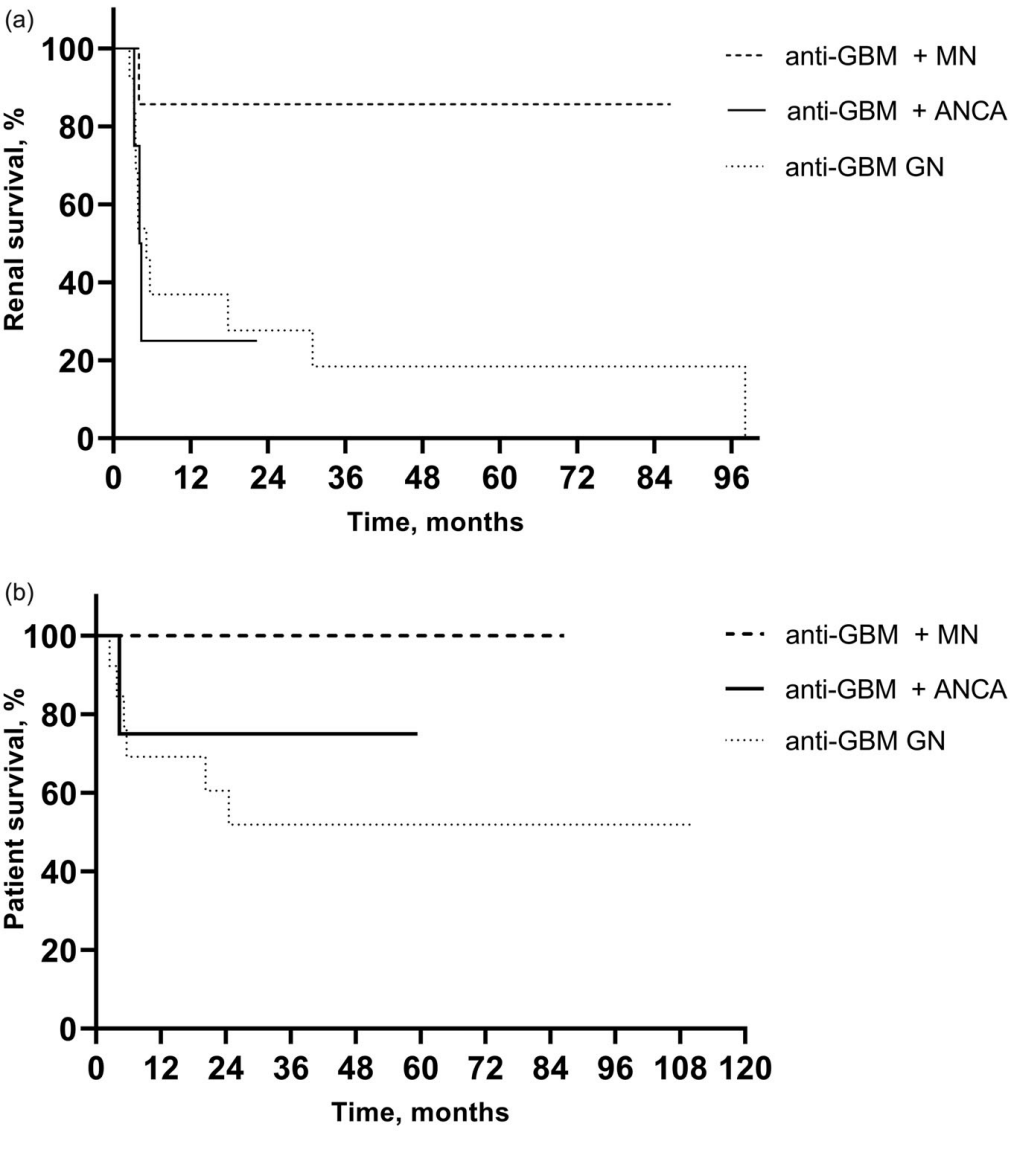
Renal and patient survival analysis of anti-GBM disease with and without MN or ANCA. (A) Renal survival among three groups. (B) Patient survival among three groups. GBM: glomerular basement membrane; GN: glomerulonephritis; MN: membranous nephropathy; ANCA: antineutrophil cytoplasmic antibodies.

GCs, CYC, and PP were the primary treatment modalities for anti-GBM disease with or without ANCAs or MN (Table 4). The proportion of patients requiring hemodialysis (HD) at the first visit was 28.6% (2/7) in the associated MN group, 66.7% (4/6) in the + ANCA group, and 69.2% (9/13) in the anti-GBM disease group, with no significant difference (*p* = .226). Renal survival at 1 year was 85.7% (6/7) in the anti-GBM + MN group, higher than the 66.7% (2/3) in the anti-GBM + ANCA group and the 55.6% (5/9) in the anti-GBM disease group, though with no significant difference (*p* = .208). A total of 85.7% (6/7) of patients showed improvement in eGFR and 14.3% (1/7) of patients depended on HD in the associated MN group. By the end of follow-up, the proportion of patients entering ESRD was 14.3% (1/7) in the associated MN group, which was significantly lower than that in the other two groups (75.0% in the + ANCA group, 3/4, *p* = .041; 84.6% in the anti-GBM GN group, 11/13, *p* = .004). Regarding patient survival, 100% of those in the anti-GBM + MN group survived, which was not significantly different from the other groups (*p* = .062). As depicted in Figure 1, renal function outcome in the anti-GBM + MN group was better than that in the anti-GBM disease group, and the difference was statistically significant (*p* = .019). By the end of follow-up, the renal survival rate was still higher than 50% in the anti-GBM + MN group. The renal median survival time was 4.2 months in the anti-GBM + ANCA group and 5.1 months in the anti-GBM disease group, with no significant difference in renal prognosis between these two groups (*p* = .877) or in patient survival among the three groups (*p* = .164).

**Table 4.** Treatment and outcome of anti-GBM patients with and without MN or ANCA.

	Anti-GBM + MN (*n* = 7)	Anti-GBM + ANCA (*n* = 6)	Anti-GBM GN (*n* = 13)	*p* value
Treatment, % (*n*)				
GCs, % (*n*)	–	–	7.7 (1/13)	–
GCs + Pulse, % (*n*)	–	–	7.7 (1/13)	–
GCs + PP, % (*n*)	–	16.7 (1/6)	–	–
GCs + Pulse + PP, % (*n*)	–	33.3 (2/6)	7.7 (1/13)	.185
GCs + Pulse + CYC, % (*n*)	28.6 (2/7)	16.7 (1/6)	7.7 (1/13)	.532
GCs + PP + CYC, % (*n*)	14.3 (1/7)	–	–	–
GCs + Pulse + PP + CYC, % (*n*)	57.1 (4/7)	33.3 (2/6)	53.8 (7/13)	.769
HD only, % (*n*)	–	–	15.4 (2/13)	–
HD at presentation, % (*n*)	28.6 (2/7)	66.7 (4/6)	69.2 (9/13)	.226
Renal survival at 1 year, % (ev/av)	85.7 (6/7)	66.7 (2/3)	55.6 (5/9)	.208
Patient survival at 1 year, % (ev/av)	100.0 (7/7)	75.0 (3/4)	69.2 (9/13)	.062
Progression to ESRD, % (ev/av)	14.3 (1/7)	75.0 (3/4)	84.6 (11/13)	.002^a^
Patient survival, % (ev/av)	100.0 (7/7)	75.0 (3/4)	53.8 (7/13)	.077
Follow-up time, month (median)	21.6	13.3	24.5	.547

GBM: glomerular basement membrane; GN: glomerulonephritis; MN: membranous nephropathy; ANCA: antineutrophil cytoplasmic antibodies; GCs: glucocorticoids; Pulse: pulsed methylprednisolone; PP: plasmapheresis; CYC: cyclophosphamide: HD: hemodialysis; ESRD: end-stage renal disease.

ev: of patients with event. av: of patients with data available.

^a^Anti-GBM + MN versus anti-GBM + ANCA, *p* = .041. anti-GBM + MN versus anti-GBM GN, *p* = .004. anti-GBM + ANCA versus anti-GBM GN, *p* = .108.

## Discussion

In 1958, Stanton and Tange reported nine cases of pulmonary hemorrhage and acute renal failure [20]. Goodpasture, described similar symptoms in young people who died during the influenza pandemic of 1918 and proposed ‘Goodpasture’s syndrome’ [21]. Further studies found that 50% of these patients were confined to the kidney [22]. In uncommon cases, anti-GBM disease can be associated with other nephropathies, with MN involvement being rare.

In this study, we analyzed seven cases of this rare entity: anti-GBM + MN. They showed chronic onset and no pulmonary lesions; serum C3 and Hb levels of anti-GBM + MN were higher than those in anti-GBM GN patients. This indicates that development is slower and immune activation degree is weaker in anti-GBM + MN than in anti-GBM disease.

In the cases with associated MN, proteinuria was in the level of 1.08 (0.40, 2.64) g/24 h, which was lower than typical MN. After excluding factors of immunosuppressive treatment, PP or secondary causes, we assume that it may be one of the special features of the atypical anti-GBM GN with associated MN. Basford et al. [23] analyzed anti-GBM + MN cases in different order of onset. It was found that patients with prior onset of MN were all middle-aged and elderly. The renal manifestations were mainly edema, and the prognosis was relatively poor. Patients with prior onset of anti-GBM GN were younger and had hematuria, whose prognosis was better. Patients with simultaneous anti-GBM GN and MN ranged in age from 16 to 71 years, and the main renal manifestations were hematuria or edema. Half of them entered ESRD or died, and the other half were cured. In our study, the anti-GBM + MN patients were mainly middle-aged and elderly. They showed microscopic hematuria and only 14.3% had massive proteinuria. The sequence of onset seems to be related to the presentations. Whether the two nephropathies happened simultaneously or successively was uncertain in our study. And that may be one reason why they were unusual as MN lacking massive proteinuria. Moreover, 85.7% of anti-GBM + MN in our study had hypoproteinemia, which was one characteristic of typical MN. The different natural course of the patients on admission may also play a role in the phenomenon.

**Figure 2.**
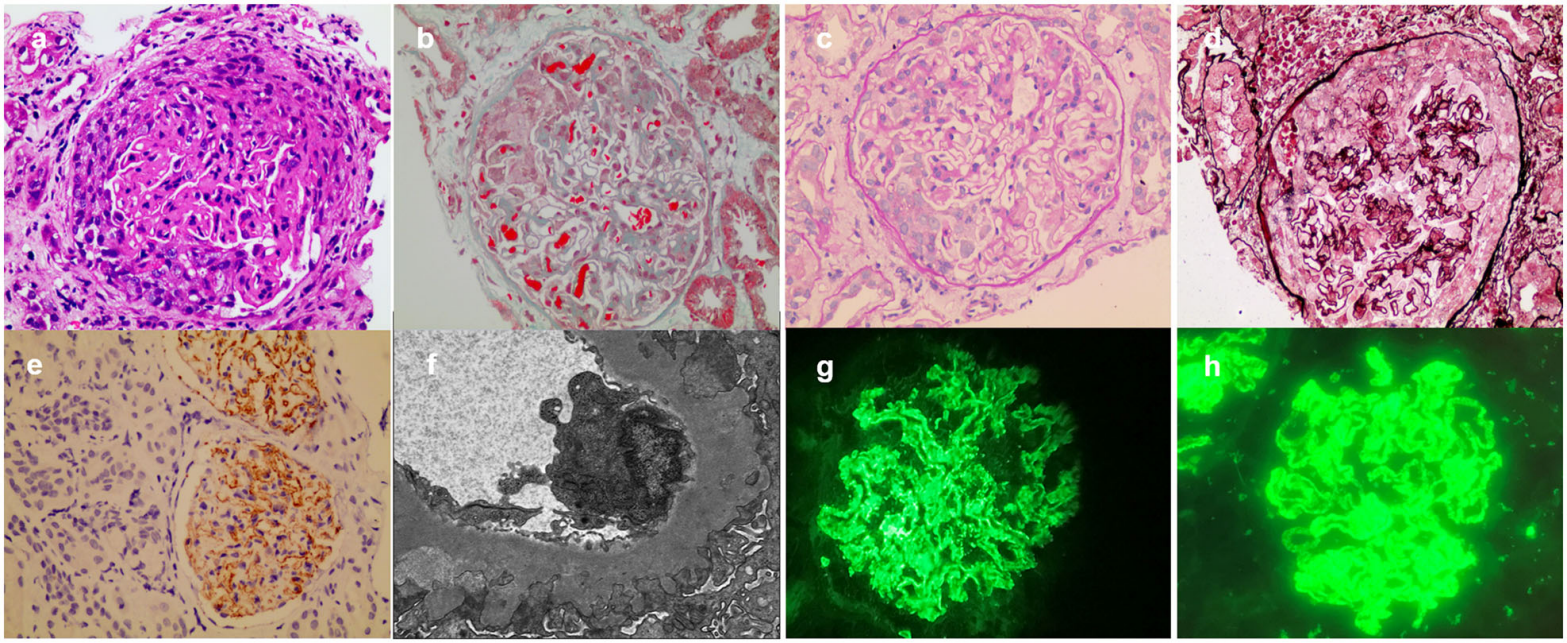
Renal biopsy specimen with anti-GBM + MN. (A)–(D) show cellular and fibrocellular crescent formation, glomerular basement membrane thickening and segmental spikes, and scattered protein deposits below the epithelium. (A) Hematoxylin-eosin staining ×400, (B) Masson trichrome staining ×400, (C) periodic acid Schiff (PAS) staining ×400, (D) PAS‑silver methenamine staining ×400, (E) immunohistochemical staining (×400) showing PLA2R positivity in subepithelial localization, (F) electronic microscopy (×10,000) showing subepithelial electron-dense deposits, (G) immunofluorescence staining (×400) showing linear and granular IgG staining along the GBM, (H) immunofluorescence staining (×400) showing granular C3 staining along the GBM.

Detailed analysis of pathological samples from seven anti-GBM + MN patients revealed all to be in the early stage of MN; IgG and C3 staining were granular or accompanied by a linear distribution along the GBM. All of the samples stained for IgG subtype were positive for IgG4. In addition, the rate of PLA2R positivity reached 66.7%. As illustrated in Figure 2, the pathological manifestations of anti-GBM + MN were represented by early MN with cellular and fibrocellular crescents. In anti-GBM disease, the IgG class of pathogenic antibodies is dominated by IgG1 and IgG3 subclasses [24,25]. We speculate that IgG4 may be related to associated MN lesions, among which the most common autoantibody, anti-PLA2R, is dominated by IgG4 [26]. Prior studies noted that anti-GBM GN with IgG4 type predominance had a favorable prognosis but those with IgG1 and IgG3 types were associated with severe renal insufficiency [24,27]. Moreover, the distribution of IgG subclasses of anti-GBM + MN was studied by Jia et al. [13]. They found that all subclasses of anti-A3(IV)NC1 antibodies could be detected in the serum of anti-GBM patients with and without MN. The levels of anti-A3(IV)NC1 IgG1, IgG2, and IgG3 in patients with MN were significantly lower than those in patients with typical anti-GBM disease. And the IgG4 subtype was 100% positive in anti-GBM + MN. The existence and function of IgG4 anti-GBM antibody need further experimental verification.

As to the association with PLA2R in anti-GBM + MN, two cases were positive for circulating anti-PLA2R and four cases were stained positively with PLA2R in renal tissue. Because the positive cases did not correspond to the same patients, 85.7% (6/7) cases were associated with PLA2R in anti-GBM + MN. This was a marked difference from previous studies. In the study of Jia et al. [13], there was no detectable response to PLA2R in the serum of anti-GBM + MN patients, and PLA2R expression was enhanced in only 25% of these patients. The exact reason for the difference has not yet been clarified by studies. We speculate that the pathogenesis of MN lesions in anti-GBM + MN has heterogeneity and might not always be associated with anti-PLA2R reactivity.

Previous studies revealed that half of patients with anti-GBM + MN were diagnosed with these two diseases simultaneously; thus, determining the development sequence is challenging [23,28]. Given that both anti-GBM and anti-PLA2R antibodies may be positive, there could be a common triggering mechanism allowing cryptic epitopes of podocyte antigen and GBM antigen to be exposed simultaneously. Cases of anti-GBM GN progressing from MN have been reported by Klassen et al., Moorthy et al., Kurki et al., and Imtiaz et al. [11,29–31]. In addition, Agodoa et al. Elder et al., Kielstein et al., and Hecht et al. reported cases of anti-GBM disease preceding MN [32–35]. Animal experiments also suggest the association. In the Heymann nephritis model, anti-GBM antibodies may assist in the formation of gp330 complexes accumulating outside the GBM [36]. Studies also indicated that granular immune deposits developed subepithelially when mice were primed with human α3(IV)NC1 [37].

Overall, evidence indicates that the coexistence of anti-GBM disease and MN is not accidental. In cases of MN preceding anti-GBM disease, we speculate that subepithelial immune complexes cause conformational changes in the quaternary structure of collagen IV, making the cryptic epitopes more accessible [38]. For anti-GBM disease preceding MN, on the one hand, GBM or other autoantigens are possibly released into circulation, and their immune complexes then deposit [39]; on the other hand, *in situ* immune complexes of anti-GBM antibody may enter the subepithelial space by capping and shedding.

Considering the previous studies and our cases, the pathology of anti-GBM + MN has the characteristics of crescents in a similar extent [12,13]. For MN complicated with crescents, ANCA was usually considered first according to previously published case series, whose positive rate was 13%–47% [40,41]. However, the combination of anti-GBM disease, although accounting for a small proportion, still cannot be ignored. Compared with MN patients, the combination of anti-GBM disease can bring an adverse impact on the prognosis [13]. Therefore, it is necessary to detect circulating anti-GBM antibody in MN + crescents patients so as to adjust the treatment regimen in time and protect the renal function.

In our study, only 14.3% of patients displayed crescentic GN. In the other cases, crescents were predominant in cellular types rather than fibrous ones. Typical anti-GBM GN has a high proportion of renal crescents reaching 90%–100%, which are usually at the same stage [42]. In ANCA-associated GN, crescents tend to be at different stages. The lower proportion of glomeruli involving crescents and the predominance of cellular types might be reasons for the better prognosis of anti-GBM + MN [12,13].

Compared with typical anti-GBM GN, it seems extraordinary that anti-GBM + MN had a low rate of AKD (28.6%) and of crescentic GN (14.3%). On the one hand, it may be one of the special “atypical” features of this disease. Most of “atypical” anti-GBM GN in previously published studies had a negative or low level of circulating anti-GBM antibodies, although they were similarly characterized by a lower proportion of crescents and better prognosis than typical anti-GBM GN [43–45]. And all the cases in our study were positive for anti-GBM. Our special cases might belong to another lineage type of ‘atypical’ anti-GBM disease which were different from the previously published atypical ones. On the other hand, it is also considered that it may be affected by associated MN. Since non-AKI patients lacked renal histopathological information at the earlier onset, the possibility of anti-GBM GN subsequently superimposing on MN cannot be excluded. And the potential effects of MN against the emergence of anti-GBM antibodies might be one reason why the course of onset and proportion of crescents were different from that of typical anti-GBM GN [12].

In this study, anti-GBM + MN patients were treated mainly with GCs (oral or combined with high-dose intravenous pulse initially) and CYC (periodic intravenous pulse therapy). Combined PP was performed to rapidly remove autoantibodies from the circulation. GCs act as potent anti-inflammatory agents, and CYC can prevent synthesis of autoantibodies. The renal survival of anti-GBM + MN was 85.7% after 1 year, which was higher than the rate in the study by Jia et al. [13]. By the end of follow-up, the proportion of patients entering ESRD in the associated MN group was significantly less than that in the + ANCA group and anti-GBM GN group. Moreover, all of the anti-GBM + MN patients survived. Survival curve analysis revealed that the outcome of renal function of anti-GBM + MN was better than that of anti-GBM disease. The better prognosis for the associated MN group is supported by the follow-up study of Jia et al. [13]. McAdoo et al. found that anti-GBM + ANCA patients had long-term renal survival, though with a high proportion of recurrence [7]. Further follow-up and larger sample sizes are needed to analyze the impact of ANCA positivity on the prognosis of anti-GBM disease.

Because our retrospective study targeted rare disease entities with small sample sizes, drawing accurate conclusions on the association is difficult. Additionally, differences in pathological lesions were not analyzed owing to the lower proportion of renal biopsies for patients with acute onset and severe conditions. Due to inaccessible frozen renal tissue, we could not perform tests of other antigens for all subjects.

In conclusion, anti-GBM + MN patients showed atypical anti-GBM GN, which had fewer crescents, slower onset, and better renal function prognosis than typical anti-GBM GN patients. Additional immune complex nephritis like MN could occur in anti-GBM disease patients, and early identification and treatment is needed for renal recovery.

## Data Availability

The data that support the findings of this study are available from the corresponding author upon reasonable request.
